# Factors predictive of extensive use of CPAP treatment in obstructive sleep apnoea

**DOI:** 10.1007/s11325-024-03146-6

**Published:** 2024-08-20

**Authors:** Fatma Doghman, Haitham Ballo, Ulla Anttalainen, Tarja Saaresranta

**Affiliations:** 1 Department of Pulmonary Diseases, Division of Medicine, Turku University Hospital, Kiinamyllynkatu 4-8, Turku, FI-20520 Finland; 2 Sleep Research Centre, Department of Pulmonary Diseases and Clinical Allergology, University of Turku, Turku, Finland; 3 Heart Center, Turku University Hospital, Turku, Finland; 4 Turku PET Centre, Turku University Hospital and University of Turku, Turku, Finland

**Keywords:** Continuous positive airway pressure, Obstructive sleep apnoea, Body mass index, Depression, Treatment

## Abstract

**Aim:**

In patients with obstructive sleep apnoea (OSA), the benefits of continuous positive airway pressure (CPAP) therapy are increased for every additional hour of daily CPAP usage. However, the data of predictors of extensive usage is scarce, if any. Therefore, we evaluated potential predictors affecting extensive treatment usage.

**Methods:**

In this retrospective study, we compiled an institutional cohort of consecutive patients diagnosed with who started CPAP therapy 1999–2022 and were included in a wireless telemonitoring system in May 2022 (*N* = 14,394). Patients using CPAP device ≥ 9 h/d were stratified into a younger (< 65 years; *N* = 124) and an older group (≥ 65 years; *N* = 131).

**Results:**

We found 255 patients (male 61%) eligible for our study, with a median age of 65 (interquartile range, IQR 55–73) years, and mean body mass index (BMI) of 36 ± 6.9 kg/m^2^. Median CPAP use was 10 h/d (IQR 10–11). BMI and depressive symptoms (DEPS) in the younger group were higher than in the older group (37.9 ± 7 vs. 34.6 ± 6.4 kg/m^2^, *p* < 0.001 and 11 (IQR 5–20) vs. 7 (IQR 5–14), *p* = 0.01, respectively). During follow-up, the BMI of the younger group increased (39.9 ± 12.5 kg/m^2^ vs. 37.9 ± 7 kg/m^2^, *p* = 0.009). DEPS values decreased in the younger group and became comparable between the groups. In multivariate models, the baseline BMI independently predicted extensive CPAP use among the younger age group, and the mask leak among the older group.

**Conclusion:**

BMI at baseline in the younger and mask leak in the older group could be independent predictive factors for extensive use of CPAP.

## Introduction

Obstructive sleep apnoea (OSA) is a common sleep-related breathing disorder. The number of OSA patients is increasing, and the vast majority are still undiagnosed. OSA affects hundreds of millions of individuals worldwide, with a prevalence of 45% considered moderate to severe [1]. Moreover, according to a recent study, the prevalence of clinically diagnosed OSA was 3.7% in the Finnish adult population, and the 1-year incidence was 0.6%^2^. Multimorbidity was present in 63% of individuals at the time of OSA diagnosis. Of those with incident sleep apnoea, 34% were heavily multimorbid, presenting with four or more comorbidities.

Continuous positive airway pressure (CPAP) is the treatment of choice for moderate to severe OSA [2, 3]. CPAP adherence remains stable in long-term follow-up but does not improve weightloss [4]. To improve OSA symptoms and possible cardiovascular and cerebrovascular outcomes, patients on CPAP therapy should use it more than 4 h per night [5]. Moreover, the clinical significance of the effect of CPAP therapy is improved for every additional hour of CPAP usage per night. However, a clear definition of optimal CPAP adherence is impossible because there are insufficient data on the relationship between usage hours and major clinical outcomes. Previous studies have focused mainly on the predictors of poor adherence to CPAP, or the optimal adherence level needed for the outcome in question [6]. In addition, predictors of extensive CPAP adherence have not been clarified. It may be speculated whether extensive use of CPAP treatment is optimal or possibly linked with health problems or issues with CPAP treatment.

Telemedicine is considered as an approach to evaluate public health challenges in chronic diseases, offering potential cost-effective management options in the long run [7]. Wireless telemonitoring represents an established option to support the routine clinical follow-up of OSA patients receiving CPAP treatment without reducing treatment efficacy or patient satisfaction [8]. This technology enables the evaluation of treatment pressures, air leaks, apnoea-hypopnoea index (AHI), and usage time and the transfer of this data to the patient’s health-care provider on a daily basis [9]. Based on our clinical experience, there is a subgroup of patients using CPAP even more than 12 h a day, but no data are available about the profile of these patients.

This retrospective observational study aims to identify potential predictors of extensive CPAP usage in OSA patients using wireless telemonitoring.

## Study design and subject selection

In this retrospective study, we compiled an institutional cohort of consecutive patients (age ≥ 18 years) diagnosed with OSA and who had started CPAP therapy (including fixed PAP and automatic PAP) between 1999 and 2022. Patients were diagnosed with cardiorespiratory polygraphies mainly done at home. These sleep studies were scored according to the rules of the American Academy of Sleep Medicine being valid at the time of performed sleep studies. The telemonitoring (ResTraxx Online System™ or Airview™, ResMed, Sydney, Australia) included also patients who started CPAP treatment during 1999–2012, and were connected to the telemonitoring system in 2013. As this retrospective study included anonymized register data, no ethics committee approval was required according to Finnish legislation. Patients were eligible for our analysis if their average daily CPAP use was ≥ 9 h/day, and they were included in the wireless telemonitoring system (Airview™, ResMed, Sydney, Australia) in May 2022 (*N* = 14,394).

### Data collection

Patients were identified based on the device number. Patients’ demographic data was extracted from Auria Clinical Informatics from Turku University Hospital. This data included age, gender, body mass index (BMI), the severity of sleep apnoea in a diagnostic cardiorespiratory polygraphy, comorbidities at diagnosis, and mental, behavioural, and neurodevelopmental disorders. The scores from subjective sleepiness measured by the Epworth Sleepiness Scale (ESS) [10] (10 points or more is interpreted as excessive daytime sleepiness), psychological distress measured by the General Health Questionnaire 12 (GHQ-12) [11] (3 points or more, the test is “positive” for psychological distress), insomnia symptoms with insomnia severity index (ISI) [12] (15 points or more is interpreted as positive for insomnia symptoms) and depressive symptoms screened by the DEPS scale [13] were included in the collected data. DEPS is a standard self-rating depression questionnaire commonly used in Finland for > 15 years and consists of 10 items that cover the core of depression symptoms. The score ranges between 0 and 30, and scores ≥ 9 suggest depression [13]. Furthermore, CPAP registry data (CPAP use, pressure setting, residual AHI and mask leakage) were obtained from the telemonitoring system. We used the extracted clinical information to evaluate the relationship between extensive CPAP usage and multimorbidity. Multimorbidity was defined as two or more chronic diseases patients had during data collection. The chronic diseases included in this study were hypertension, diabetes mellitus, chronic obstructive pulmonary disease (COPD), morbid obesity with alveolar hypoventilation, ischemic heart diseases, interstitial pulmonary diseases, depression, and any cancer.

Telemonitoring data of all included patients were re-evaluated during the last six months. The patients were divided into two groups for descriptive purposes. Since retirement has an impact on different risk factors for chronic diseases [14, 15], the included patients were stratified according to statutory retirement age in Finland into two groups: before retirement (< 65 years group) and at retirement and after (≥ 65 years group) [16].

### Statistical analysis

A Shapiro-Wilk W-test was used to test the distribution of the data. A two-sample t-test or nonparametric Mann-Whitney test was used to compare the continuous variables and variables expressed as mean ± standard deviation (SD) or median and interquartile range (IQR). Categorized variables were analyzed using a chi-square test and expressed as numbers and percentages. The changes in continuous variables during follow-up were tested using paired t-test or Wilcoxon signed ranked test. Correlations are performed to analyze the relations between the variables using the Spearman correlation coefficient. Multiple variables regression was used to determine the effect of CPAP treatment. P values < 0.05 were considered statistically significant. Statistical analyses were done using SPSS v.29.01 (IBM Corporation, New York, USA).

## Results

With Insight software™ (ResMed, Sydney, Australia,) the final number was 255 patients eligible for our study (1,9% of those using CPAP), after excluding the patients with missing data in one or more items described above. Of the eligible patients, 124 were younger than 65 years and 131 were 65 years or older. Median age was 65 (IQR 55–73) years, 61% were male, mean BMI 36 ± 6.8 kg/m^2^, and median of average daily CPAP usage was 10 (IQR 10–11) hours/day. There were no significant differences between the two age groups regarding demographic and clinical variables, except that obesity (BMI) was higher in the younger group, and hypertension was more common in the older group (Table 1).

**Table 1:** Basic characteristics

	Total	< 65 Years	≥ 65 Years	
	(*n* = 255)	(*n* = 124)	(*n* = 131)	
Age, years	65 (55,73)	55 (47,60)	73 (69,79)	< 0.001
Male (%)	155 (61)	76 (61)	79 (60)	0.9
BMI (kg/m^2^)	36 ± 6.9	37.9 ± 7	34.6 ± 6.4	< 0.001
CPAP usage (hours/day)	10 (10–11)	10 (10–11)	10 (10–11)	0.2
Mask leak (L/min)	6 ± 12	5.9 ± 11	6.1 ± 12.2	0.9
Hypertension n (%)	153 (60)	65 (25.5)	88 (34.5)	0.02
Cardiovascular disease n (%)	12 (4.7)	5 (4)	7 (5.3)	0.4
DM n (%)	81 (32)	35 (14)	46 (18)	0.6
COPD n (%)	13 (5.1)	9 (3.5)	4 (1.6)	0.08
Depression n (%)	81 (32)	38 (15)	43 (17)	0.7
Cancer n (%)	20 (7.8)	7 (2.7)	13 (5.1)	0.2

Values are presented as mean and SD, median interquartile range, or number and percentage. BMI, body mass index; DM, diabetes mellitus; COPD, chronic obstructive pulmonary disease

At the baseline, there were no statistical differences among the groups in terms of subjective sleepiness (ESS), sleep apnoea severity (AHI), psychological distress (GHQ-12) or insomnia symptoms (ISI). However, the baseline BMI and depressive symptoms (DEPS) in the younger group were significantly higher than in the older group (37.9 ± 7 kg/m^2^ vs. 34.6 ± 6.4 kg/m^2^, *p* < 0.001 and 11, (IQR 5–20) vs. 7, (IQR 5–14), *p* = 0.01, respectively). Compared with baseline, after CPAP treatment, significant decreases in AHI and ESS scores were observed for both groups, while DEPS and ISI scores were significantly changed only in the younger group. Moreover, after CPAP treatment, the BMI of the younger group increased dramatically from 37.2 ± 7.3 to 39.9 ± 12.5, *p* = 0.006 (78 patients, 63% of the young population). In addition, it became higher than the BMI of ≥ 65 years group after six months of CPAP treatment (*p* < 0.001). Conversely, DEPS scores after CPAP treatment were comparable among the two groups (9.2 (IQR 5–20) vs. 6 (IQR 3–10), *p* = 0.6). (Table 2)

**Table 2:** Sleep parameters for the total group, < 65 years, and ≥ 65 years groups at the baseline and at the six months’ follow-up

	Baseline	Follow-up	
< 65 years			
(*N* = 124)			
BMI (kg/m^2^)	37.9 ± 7*	39.9 ± 12.5	0.009
AHI	34 (18–54)	2.3 (1–3)	< 0.001
ESS score	8.4 (4–13)	4 (1–7)	< 0.001
GHQ-12 score	5.1 (1–9)	2 (0–8)	0.22
DEPS score	12.5 (5–20) *	5 (2–15)	0.02
ISI score	16 (8–19) *	8 (4–10)	0.03
≥ 65 years			
(*N* = 131)			
BMI (kg/m^2^)	34.6 ± 6.4	31.5 ± 6.3	0.2
AHI	37 (21–59)	3 (1–4)	< 0.001
ESS score	7 (4–10)	3 (1–5)	< 0.001
GHQ-12 score	2 (0–5)	1 (0–6)	0.8
DEPS score	7 (4–13)	6 (3–10)	0.2
ISI score	7 (5–14)	5 (3–9)	0.3

Values are presented as mean and SD, or median and interquartile range. BMI body mass index, AHI apnoea-hypopnoea index, ESS Epworth Sleepiness Scale, GHQ12 General Health Questionnaire 12-item version, DEPS depressive symptoms scale, and ISI insomnia severity index

* vs. ≥ 65 years, *p* < 0.05

**Fig. 1:**
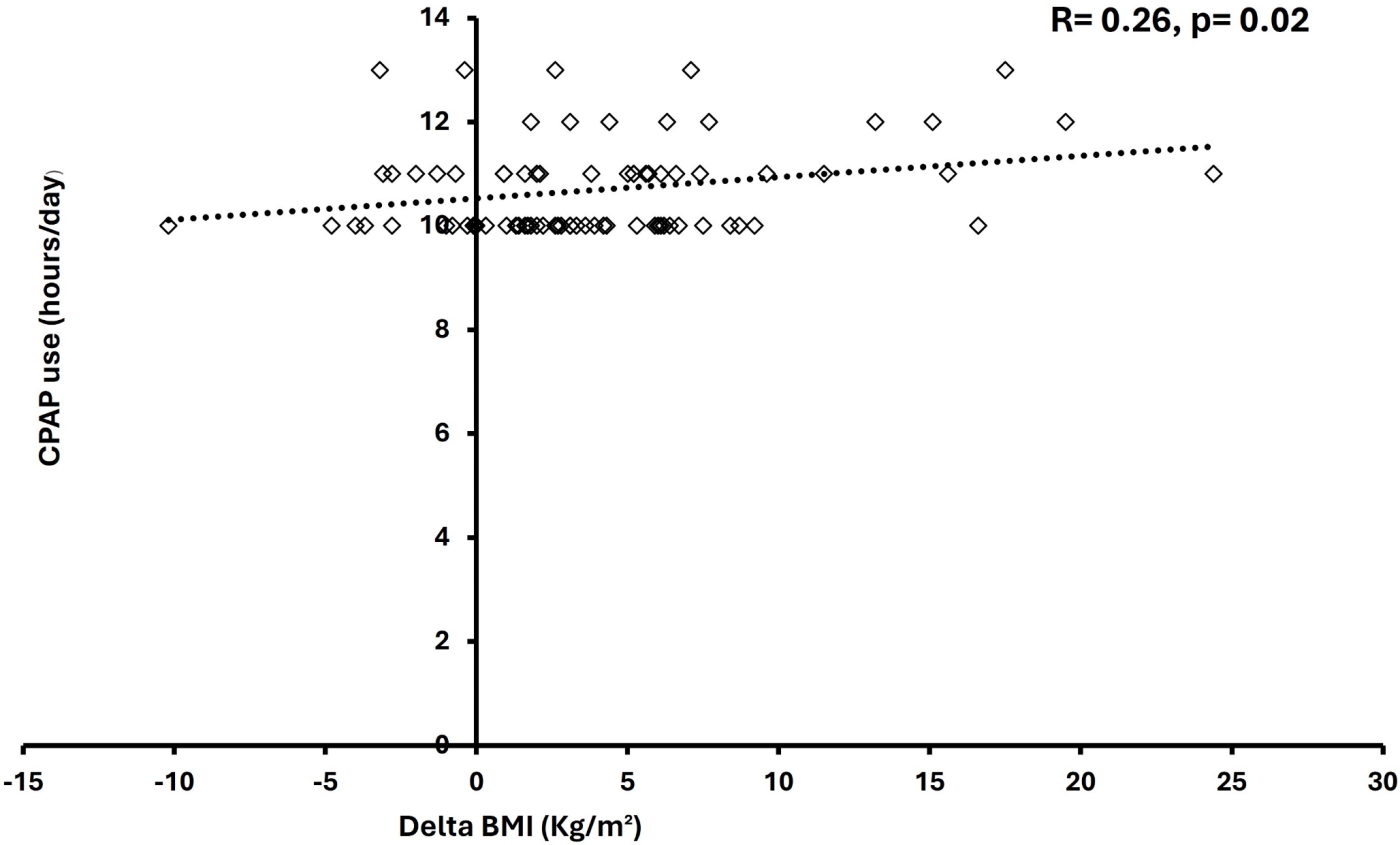
Scatter diagram of correlation analysis between BMI and extensive CPAP daily use for < 65 years group

**Fig. 2:**
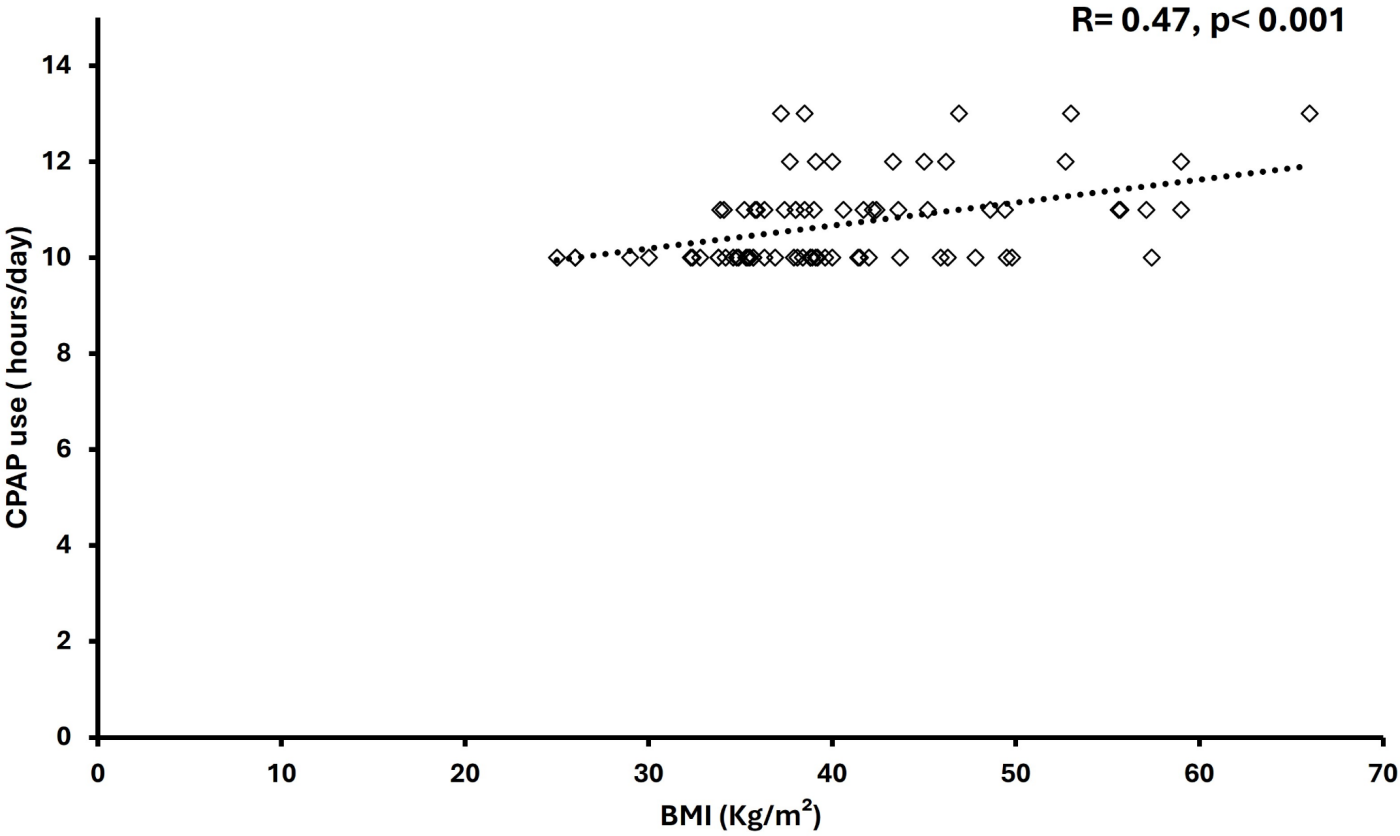
Scatter diagram of correlation analysis between delta BMI and extensive CPAP daily use for < 65 years group

**Fig. 3:**
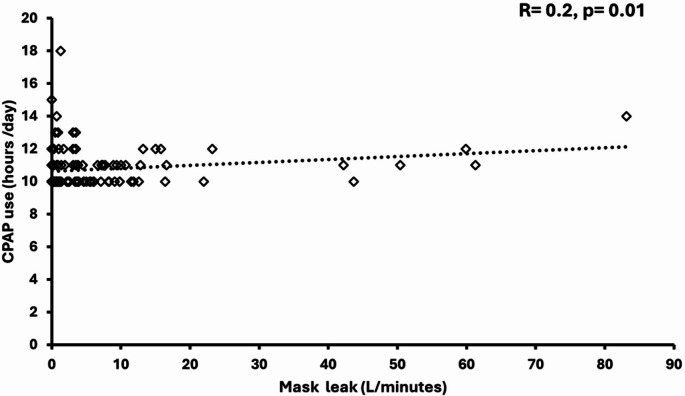
Scatter diagram of correlation analysis between mask leak and extensive CPAP daily use for ≥ 65 years group

The Spearman correlation test determined the relationship between variables and the extensive CPAP usage. There was a significant correlation between average extensive CPAP use and BMI values that were measured at follow-up in the younger group (*r* = 0.047, *p* < 0.001); increasing BMI correlated with higher CPAP usage hours (Fig. 1). Furthermore, there was a significant correlation between the average extensive CPAP usage and differences in BMI (delta) at baseline and follow-up (*r* = 0.26, *p* = 0.02) (Fig. 2). However, no correlations existed between the average extensive CPAP usage and other CPAP treatment variables. There was a significant correlation between average extensive CPAP usage and mask leak in the older group (*r* = 0.2, *p* = 0.01) (Fig. 3). Conversely, the older group had no correlations between CPAP usage and treatment variables.

In univariable regressions for the younger group, only baseline BMI (β = 0.261; 95% confidence interval [CI] 0.12, 0.16]; *P* = 0.004) and AHI (β = 0.01; 95% CI [-0.1,0.04)]; *P* = 0.004) predicted extensive CPAP usage. In the older group, the univariable analysis showed that baseline BMI (β = 0.061; 95% CI [-0.02, 0.045)]; *P* = 0.001), AHI (β = 0.089; 95% CI [0, 0.015)]; *P* = 0.01), and mask leak (β = 0.192; 95% CI [0,0.34)]; *P* = 0.03), could predict the extensive CPAP usage. However, in multiple regression analysis, baseline BMI (β = 0.277; 95% CI [0.14,0.65)]; *P* = 0.003) and mask leak (β = 0.199; 95% CI [0, 0.37)]; *P* = 0.04) were only independent predictors for average daily usage of CPAP in the younger and the older group, respectively. Results of the univariable and multiple regressions are shown in Table 3.

**Table 3:** Univariate and multiple regression analysis of the possible predictors of the mean CPAP daily use in < 65 years and ≥ 65 years groups

	Univariate regression		Multiple regression	
	ß Coefficient (95% CI)	*p*	ß Coefficient (95% CI)	*p*
< 65 years				
Predictors				
AHI	0.01 (-0.1,0.04)	**0.04**		
BMI	0.261 (0.12,0.16)	**0.004**	0.277 (0.14,0.65)	**0.003**
Mask leak	0.001 (-0.16,0.62)	0.9		
≥ 65 years				
Predictors				
AHI	0.089 (0, 0.015)	**0.01**		
BMI	0.061 (-0.02,0.045)	**0.001**		
Mask leak	0.192 (0,0.34)	**0.03**	0.199 (0,0.37)	**0.04**

BMI: body mass index, AHI: apnoea-hypopnoea index

## Discussion

This is the first study to assess predictors of extensive CPAP usage in patients using wireless telemonitoring. To our knowledge, no previous publications are focusing on the “heavy users” in particular, i.e. patients using CPAP therapy 9 h/day or more on average. The main findings of our study were that although CPAP usage hours were similar in both age groups, the BMI was higher among the younger group than the older group at the beginning of the treatment and augmented over the course of treatment. Moreover, the depression scores were higher at the beginning and improved significantly after the treatment in the younger group, whilst in the older group, there was no significant improvement after the treatment. The BMI and mask leak were independent predictors of extensive CPAP usage in the younger and older groups, respectively.

### BMI and extensive CPAP usage

Obesity is one of the critical factors for OSA, and many studies have consistently found an association between gain of weight and the risk of OSA [17]. The effects of CPAP on BMI and weight are unclear. However, some studies have linked CPAP therapy to reduced resting metabolic rate due to the hormonal impacts possible resulting the weight gain [17]. Previous studies assessed the effect of CPAP on body weight in OSA patients. Quan et al. evaluated the impacts of CPAP treatment in OSA patients for six months in a randomized controlled multicentre trial. They demonstrated that CPAP treatment was associated with weight gain [18]. The weight change was associated with CPAP adherence with each hour per night of CPAP usage associated with a mean 0.42 kg increase in weight over six months. However, the study could not observe any associations between the CPAP treatment and related factors such as age, gender, race, and baseline BMI.

Furthermore, Redenius et al. [19], in a retrospective study of 183 OSA patients treated with CPAP extended to 14 months, showed that women were more likely to gain weight after CPAP usage. Our study has a considerably larger and more extended follow-up period and, therefore is more likely to detect precisely the factors that impact CPAP use. Our findings are statistically consistent with those of Myllylä et al. [4], who evaluated OSA patients who used CPAP treatment for more than six years in a big cohort study (1000 patients), and the main finding was that weight gainers after CPAP treatment were significantly younger and had severe obesity at the beginning of the treatment. Inflammatory cytokines, tumor necrosis factor-α (TNFα) and interleukin-6 (IL-6), both of which produce sleepiness and fatigue, are elevated in OSA and obesity, which might lead to extensive usage of CPAP therapy [20]. In addition to hypercytokinemia, younger patients are likely to have higher work load and other coincident demands of daily life compared to older patients, which might explain the different effect of obesity between the age groups.

### Daytime sleepiness and CPAP

OSA patients suffer excessive daytime sleepiness, which can negatively affect daily functioning, cognition, mood, and other aspects of well-being [21]. The Epworth Sleepiness Scale (ESS) is often used to assess daytime sleepiness, which is considered to be one of the most common symptoms related to OSA [22]. Successful CPAP treatment for OSA in adherent patients often leads to symptom relief within a few weeks [23]. However, sleepiness persists in 5–9% of OSA patients despite CPAP treatment [5, 24, 25]. Furthermore, ESADA, a prospective multicentre study involving over 30,000 suspected OSA patients to explore predictors of persistent residual excessive daytime sleepiness among many factors, concluded that hours of CPAP use and length of follow-up are the significant predictive factors of residual excessive daytime sleepiness [26]. Our study showed a beneficial effect of CPAP therapy on daytime sleepiness in the long term, and significant decreases in ESS scores were observed in all groups. Our results are in line with the Avlonitou et al. study assessing the quality of life after six months for 50 OSA patients using CPAP treatment. They demonstrated that OSA patients who adhere to night-time CPAP therapy significantly improve their daytime sleepiness [27].

### Depression and CPAP

Doctor diagnosed depression was highly prevalent (30%) in our cohort of extensive CPAP users, and nearly threefold compared to that of the Finnish nationwide cohort of OSA patients [28]. Our study showed that the younger group’s DEPS score significantly declined after extensive CPAP treatment compared with their baseline score. However, the DEPS score in the older group was unremarkable at baseline and did not change after the CPAP treatment. DEPS score is an established depression questionnaire in Finland for 25 years and is recommended to identify the severity of depression in primary care patients [29]. Previous study [30] evaluated depression scores at follow-up after CPAP treatment in the group at an age comparable to the age of the younger group of our study (55 years). It showed the improvement in the depression scores was dependent on CPAP adherence. The connection between CPAP treatment and the improvement of depression symptoms is uncertain. Nevertheless, previous studies have identified that the reversal in various pathophysiological mechanisms, such as apnoea-induced sleep fragmentation, excessive daytime sleepiness, and decreased grey matter due to hypoxemia, were associated with CPAP treatment [30]. In contrast, a previous multicentre randomized controlled trial evaluated the effect of CPAP treatment in elderly patients (over 70 years old) with moderate OSA regarding quality of life. It showed there was no effect of CPAP treatment on the degree of depression or anxiety [31]. Our results extend with these observations by showing that CPAP use has a significant impact on the depression of the younger group but not the older group.

### Mask leak and extensive CPAP usage

In our study, there was a weak correlation between extensive CPAP usage and mask leak in the older group, and mask leak was considered an independent predictor for extensive CPAP use in the older group. A previous large retrospective study evaluated the impact of age on CPAP use and suggested that the increase of the mask leak with CPAP use maybe explained by increased CPAP use with increasing age [32]. However, in our results, the mask leak was very low, and there was no significant difference between the younger and older groups. Furthermore, previous study evaluated the predictors of CPAP compliance in OSA patients with metabolic syndrome for eight weeks and showed that high mask leak was the only independent predictor of CPAP compliance [33].

Our study has some limitations. First, it was a unicentric retrospective study conducted on a moderate number of Caucasian patients. Secondly, even though we have identified a potentially large patient population, only a small number of patients were finally included in this study. Due to missing values, we did not evaluate the waist circumference for all patients or include it in this study. Furthermore, the sample size of patients with cardiovascular disease was small and lacked the statistical power to assess the impact of extensive CPAP use on cardiovascular disease. Moreover, we did not consider other factors, such as the type of mask (oronasal mask/ nasal mask) or CPAP devices or sedative hypnotics, that may influence the predictive value of CPAP usage. Although we evaluated the predictiveness of extensive CPAP usage by dividing the group study according to the pension age, we did not assess the socioeconomic and personality factors that could affect CPAP usage, like employment status and education, especially among the younger group [34]. Our study did not evaluate the adverse effects of extensive CPAP usage. Not all included patients were evaluated by ISI score because it has only recently used in our centre. Finally, we did not have a matched group of patients using CPAP less than 9 h, and therefore cannot compare the predictors of CPAP usage over the entire range usage hours.

## Conclusion

In our practice, extensive CPAP usage is associated with weight gain in patients with OSA less than 65 years old already during the first months of CPAP treatment. Furthermore, our data indicate that BMI among the younger patients and mask leak in the older ones could be predictive factors for extensive usage of CPAP. Further prospective multicentre studies are recommended to evaluate predictive factors for extensive usage of CPAP.

## Data Availability

This manuscript has no associated data.
